# Biomarkers in Immunotherapy-Based Precision Treatments of Digestive System Tumors

**DOI:** 10.3389/fonc.2021.650481

**Published:** 2021-03-11

**Authors:** Zhu Zeng, Biao Yang, Zhengyin Liao

**Affiliations:** ^1^Department of Abdominal Oncology, West China Medical School, West China Hospital, Sichuan University, Chengdu, China; ^2^Department of Gastroenterology, West China Hospital, West China Medical School, Sichuan University, Chengdu, China

**Keywords:** biomarkers, programmed death-ligand 1, tumor mutational burden, microsatellite instability, circulating tumor DNA

## Abstract

Immunotherapy, represented by immune checkpoint inhibitors (mainly referring to programmed death-1 (PD-1)/programmed death-ligand 1 (PD-L1) blockades), derives durable remission and survival benefits for multiple tumor types including digestive system tumors [gastric cancer (GC), colorectal cancer (CRC), and hepatocellular carcinoma (HCC)], particularly those with metastatic or recurrent lesions. Even so, not all patients would respond well to anti-programmed death-1/programmed death-ligand 1 agents (anti-PD-1/PD-L1) in gastrointestinal malignancies, suggesting the need for biomarkers to identify the responders and non-responders, as well as to predict the clinical outcomes. PD-L1expression has increasingly emerged as a potential biomarker when predicting the immunotherapy-based efficacy; but regrettably, PD-L1 alone is not sufficient to differentiate patients. Other molecules, such as tumor mutational burden (TMB), microsatellite instability (MSI), and circulating tumor DNA (ctDNA) as well, are involved in further explorations. Overall, there are not still no perfect or well-established biomarkers in immunotherapy for digestive system tumors at present as a result of the inherent limitations, especially for HCC. Standardizing and harmonizing the assessments of existing biomarkers, and meanwhile, switching to other novel biomarkers are presumably wise and feasible.

## Introduction

As mentioned in real-world investigations, the application of immunotherapy has obtained great success in multiple tumor types, increasingly shifting the conventional treatment paradigm to precision and individual medicine (1–3). More recently, the immune checkpoint inhibitors (ICIs) also underlined promise in digestive system tumors [including gastric cancer (GC), colorectal cancer (CRC), and hepatocellular carcinoma (HCC)] with regard to the latest data reported in the 2020 American Society of Clinical Oncology (ASCO) and European Society of Oncology (ESMO) meetings (4, 5). The routine regimens of fluoropyrimidines, platinum, and anthracyclines did improve the clinical outcome in these tumors, but the prognosis remained poor (6). Unlike chemotherapy, immunotherapy principally reactivates or remobilizes the autoimmune system and is characterized by longer-duration and less-susceptibility, thus occupying more space in the clinic. In detail, the agents of pembrolizumab and nivolumab have gradually revolutionized the status of traditional therapies (chemotherapy) in advanced HCC, GC, or CRC with specific gene mutations, thereby facilitating rapid approval by the Food and Drug Administration (FDA) (7).

Given the fact that the response rate substantially varies across tumor types, and similarly, patients with the same malignancy could exhibit different responses when treated with anti-programmed death-1/programmed death-ligand 1 (anti-PD-1/PD-L1) or anti-cytotoxic T lymphocyte antigen 4 (anti-CTLA-4) inhibitions, utilizing biomarkers to identify and select who are more likely to respond and who are unlikely to respond, which further contributes to the patient-stratification, is extensively urgent. In fact, numerous studies have highlighted potential predictive biomarkers and also made some progress. Taking the PD-L1 expression (which is the most studied biomarker now) as an example, in the KEYNOTE 590 trial which evaluated the efficacy of pembrolizumab in esophageal cancer (EC), an improved outcome was obviously seen in the population with PD-L1-positive than that in those with PD-L1-negative (8). However, the results of the KEYNOTE 061 study that involved pembrolizumab in GC failed to reach a significant difference in survival (9). Even equally adopting the anti-PD-1 agent, distinct response rates are observed in EC and GC. Furthermore, the use of other biomarkers, such as tumor mutational burden (TMB), microsatellite instability (MSI), and circulating tumor DNA (ctDNA), have been studied, and no agreement has been reached (10–12).

One major limitation of these biomarkers during detection is that the definition of the corresponding cut-off values, especially for PD-L1 and TMB, remains unclear. In addition, the diversity of testing methods also results in discrepancies. Hence, it is established that there are still no perfect and sufficient immunotherapy-related biomarkers that can be widely used in pan-tumor types. Of note, further explorations are required to focus on the novel biomarkers, including the CD8^+^ tumor infiltrating lymphocytes (TILs) (13), polymerase epsilon (POLE) variation (14), and DNA methylation (15, 16).

In fact, no similar literature which simultaneously investigated and compared the immunotherapy-related biomarkers in digestive system tumors was previously reported. However, attention ought to be paid, since these biomarkers could guide the therapeutic decision-making just like targeted therapies. And another purpose is to understand them specifically and to further improve their detection methods. To provide several strategies in the clinic, we summarized the accessible biomarkers in this paper and then listed other novel molecules as well. Furthermore, it may help to make choices by comparing the available testing techniques and assays in the clinic.

## Programmed Death-Ligand 1Expression as a Biomarker

Previous findings suggesting a latent relationship among the PD-L1 levels, immunotherapy, and clinical outcomes have contributed to the widespread interest in PD-L1 as a predictive biomarker (17, 18). At present, a confirmed positive-related correlation of PD-L1expression and the efficacy of anti-PD-1/PD-L1 inhibitors have been observed in non-small cell lung cancer (NSCLC) and melanoma; yet, whether a similar relationship would exist in digestive tumors is still unclear. Those patients with PD-L1 positive presumably present a lower response or even a non-response to PD-1/PD-L1 blockades, while those PD-L1-negative participants may be benefited. Likewise, taking the KEYNOTE 062 trial (NCT02494583) in GC, for example, no differences were statistically seen when comparing patients with lower-expression PD-L1 to those with higher-expression (19). In addition, in the CheckMate 040 study in HCC, there might be no obvious correlation between PD-L1 and local tumor control (20). However, the REGONIVO trial involving CRC indicated improved efficacy as PD-L1 levels increased (21). Specifically, it may be due to the absence of PD-L1's accuracy in separating potential candidates or non-responders. Therefore, subsequent explorations highlighting the predictive value of PD-L1, including the standardization and reproducibility, are required.

### The PD-1/PD-L1 Signaling Pathway

**Figure 1:**
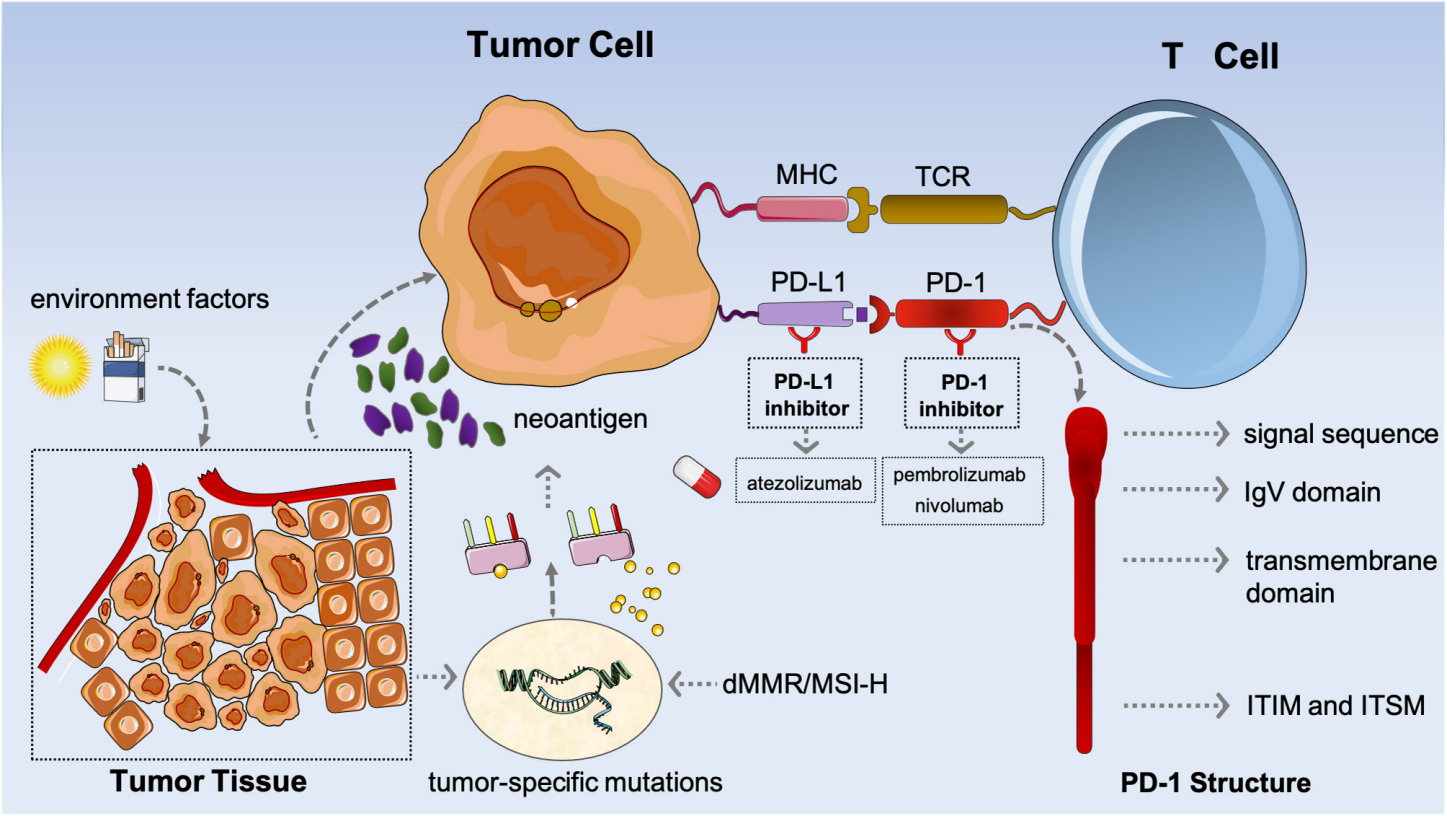
The mechanism of potential biomarkers [programmed death-ligand 1 (PD-L1)], tumor mutational burden (TMB), and microsatellite instability high or deficiency of MMR (MSI-H/dMMR) in immunotherapy.

Of note, PD-1/PD-L1 signaling is a well-recognized pathway responsible for tumor-specific immune escape; also, PD-1, a type I transmembrane glycoprotein which belongs to the CD28 superfamily, is a critical immune checkpoint and plays the role of an immunosuppressive molecule (22, 23). The typical structure of PD-1 has an extracellular immunoglobulin variable (IgV) region, a hydrophobic transmembrane domain, and an intracellular domain (Figure 1) (24, 25). Two independent tyrosine residues, which participate in forming immunoreceptor tyrosine-based inhibitory motif (ITIM) and immunoreceptor tyrosine-based switch motif (ITSM), are located at the tail of the intracellular region (25, 26). Both PD-L1 (B7-H1) and programmed death-ligand 2 (PD-L2, B7-DC) are both its respective ligands (25, 27).

Evidence has confirmed the role of PD-1 as a negative regulator on T-cell activation (28, 29). As a whole, PD-1 is expressed on the surface of the activated T cells and B cells, the natural killer (NK) cells, as well as the dendritic cells (DCs), binding with the ligands of PD-L1/PD-L2 that are distributed on the surface of antigen-presenting cells (APCs), and then further inhibiting the hyperactivation of T cells to maintain the stability of the immune system (24, 28, 30). Co-inhibitory immune receptors [killer cell immunoglobulin-like receptor (KIR), lymphocyte-activation gene 3 (LAG 3), and CTLA-4 are also included] are involved in immune regulation along with co-stimulatory receptors and soluble immune suppressors [transforming growth factor β (TGF-β) and interleukin 6 (IL-6) are included] (30, 31).

When the tumor occurs, a high-level expression of PD-1 in the infiltrating T cells is induced through the microenvironment, whereas a corresponding high expression of PD-L1/PD-L2 in tumor cells (TCs), in turn, leads to the constitutive activation of PD-1/PD-L1 and PD-1/PD-L2 signaling; then, T-cell function is attenuated or even inhibited, which prevents them from signaling to attack the TCs, potentially allowing TCs to evade immune surveillance and to further progress (Figure 1). Whereas, the representative ICIs contain anti-PD-1 antibodies (such as pembrolizumab and nivolumab) that specifically bind to PD-1 on the surface of T cells and then reinvigorate the function of tumor-specific T cells *via* obstructing the PD-1/PD-L1 or PD-1/PD-L2 signaling pathways, and anti-PD-L1 antibodies (such as atezolizumab) which only block the PD-1/PD-L1 pathway, but do not affect the PD-1/PD-L2 pathway.

### The Testing and Interpretation Methods

**Figure 2:**
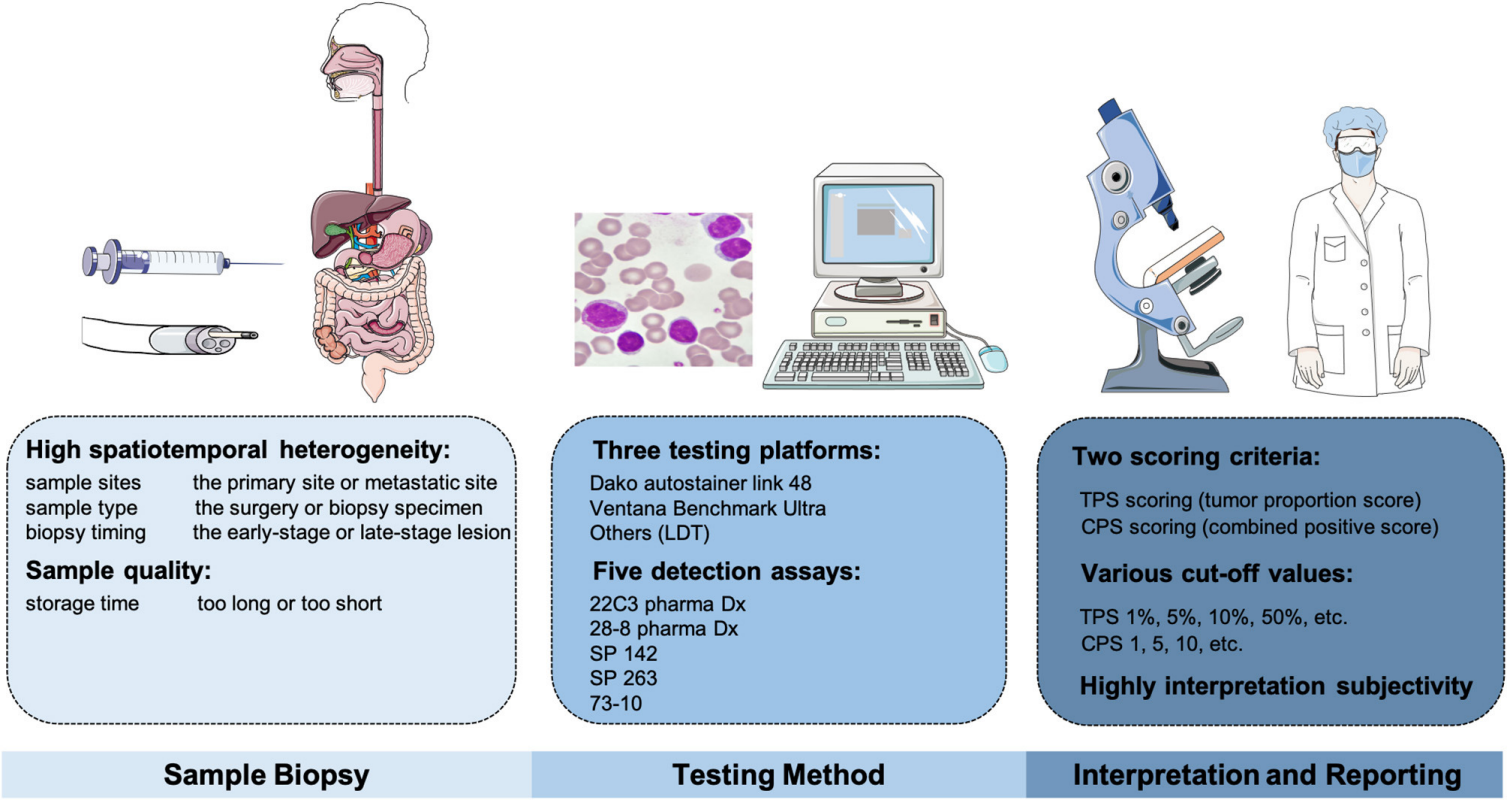
The factors influencing the accuracy of PD-L1 detection, including the sample-biopsy factors, the testing-method factors, and the interpretation factors.

Although the initial data revealed that PD-L1 expression perform poorly and is less reliable in digestive system tumor types when acting as a biomarker, no denying that multiple factors cause this phenomenon. Immunohistochemistry (IHC) is currently utilized as a PD-L1 measurement method, but standardized assays and uniform thresholds are lacking (Figure 2). FDA has approved five detection kits to be available for PD-L1 IHC staining, including the 22C3 pharma Dx, 28-8 pharma Dx, SP 142, SP 263, and 73-10 assays. These assays are mainly detected on two IHC platforms, Dako autostainer link 48 (available for 22C3, 28-8, and 73-10) and Ventana Benchmark Ultra (available for SP142 and SP 263). Meanwhile, different staining platforms are used to test various antibodies; for example, FDA approved Dako 22C3 pharma Dx PD-L1 as a companion diagnostic for pembrolizumab, and Dako 28-8 and Ventana SP142 PD-L1 was approved as complementary diagnostics for nivolumab and atezolizumab, respectively. To assess the reliability of these approaches and harmonize PD-L1, Lantuejoul et al. (32) analyzed 41 NSCLC surgical specimens *via* three platforms [Dako, Ventana, and laboratory-developed tests (LDTs) are included], which involved five IHC PD-L1 detections (22C3, 28-8, SP142, SP 263, and E1L3N assays). The staining results in TCs and immune cells (ICs) suggested a high-consistency in 28-8, 22C3, and SP 263, as well as a dynamic change in LDTs.

Another cause is due to the differences of interpretation methods adopted by these platforms [tumor proportion score (TPS)], which is defined as the proportion of living TCs with PD-L1 partially or completely stained for PD-L1 relative to all surviving TCs in the sample; combined positive score (CPS), which is defined as the amount of all positivity stained cells in the samples, including TCs, macrophages, and lymphocytes) (33). As a result, the cut-off value did not reach a consensus when PD-L1 present positive. For example, in some tumor types, determining a value of 10% or more in TCs is defined as PD-L1 (+) when applying the TPS criteria, such as in urothelial cancer; while some use a value of 50%, such as in NSCLC. Intriguingly, among variable investigations in GC, the cutoffs vary from CPS ≥1, ≥5 to ≥10 when selecting an appropriate threshold. Certainly, this could explain to some extent why the results were inconsistent. Mechanically, apart from TCs, PD-L1 can be expressed by ICs as well. It seems more reasonable that CPS is not limited to TCs, and ICs are comprehensively considered.

Additionally, PD-L1 expression is characterized as high spatial and temporal heterogeneity, which mainly reflects the following aspects (34). First of all, PD-L1 levels show a dynamic trend at different stages; that is to say, the assay results can be perturbed by the biopsy timing and tissue origins. Similarly, the results may be highly differentiated from the primary tumor and metastatic sites. Then, even in the same tumor tissue, different PD-L1 expressions may exist in different biopsy sites, so multiple-regional sampling is feasible. Furthermore, the pre-treatment of biopsy specimens is also crucial.

### The Clinical Utility of PD-L1 Expression in Anti-PD-1/PD-L1 Therapy

Based on these limitations, PD-L1 expression is insufficient as an independent biomarker, but its role in patient stratification should not be overlooked when receiving anti-PD-1/PD-L1 treatments, as PD-L1 indeed indicates some association with immunotherapeutic efficacy in multiple clinical trials. Moreover, it is also essential for PD-L1 expression to be standardized and universal across diverse cancer types.

#### Pembrolizumab

Pembrolizumab, an anti-PD-1 antibody with applications in digestive system tumors, particularly in EC and GC, has been extensively studied. Meanwhile, the studies involving the association between the PD-L1 status and the immunotherapeutic response is underway, with the aim of further screening out the best-responders and excluding non-responders from the whole population to maximize treatment benefits and minimize toxicities.

##### In Esophageal Cancer and GC

Actually, PD-L1 level is frequently utilized to differentiate the dominant populations in EC and GC, and as seen in the KEYNOTE series of trials, the higher PD-L1 CPS scores are followed by longer overall survival (OS) after treatment with ICIs. When mentioned the applications of ICIs in EC, pembrolizumab has been recommended as a second-line option for PD-L1-postive, advanced EC by National Comprehensive Cancer Network (NCCN) guideline (version 2019).

*In esophageal cancer*. Specifically, KEYNOTE 180 (NCT02559687) was an open-label, single-arm, phase II trial designed to evaluate the efficacy and safety of pembrolizumab monotherapy (200 mg, every 3 weeks) in advanced esophageal squamous cell cancer (ESCC)/gastroesophageal junction cancer (EGJC), totally recruiting 121 patients who previously failed at least second-line therapy (35). Notably, PD-L1 CPS ≥ 10 was defined as PD-L1-positive *via* the PD-L1 IHC 22C3 pharmDx assay and PD-L1 negative otherwise. Of the 121 participants, 58 patients (48%) were PD-L1 (+), and 63 patients (52%) were PD-L1 (–). Subgroup analysis described in this trial demonstrated that an improved median overall survival (mOS) was observed in the PD-L1-positive arm (6.3 months, 4.4–9.3) than the PD-L1 negative arm (5.4 months, 3.9–6.3), as well as a higher disease control rate (DCR) in the PD-L1 (+) group (36 vs. 25%). Subsequently, the 2018 ASCO meetings provided the 1-year follow-up data from the KEYNOTE 180 trial, with a higher overall response rate (ORR) in the PD-L1 (+) population (CPS ≥ 10) when compared with the PD-L1 (–) population (CPS <10) (4 vs. 6%). Based on KEYNOTE 180, at the 2019 ASCO meetings, Kojima et al. reported a phase III clinical trial (KEYNOTE 181, NCT02564263) to compare pembrolizumab alone with chemotherapy (paclitaxel plus docetaxel/irinotecan) when used as the second-line treatment for late-stage EC (36). A total of 628 enrolled patients were randomized to either the pembrolizumab or chemotherapy group at a ratio of 1:1. Similarly, the value of CPS 10 was determined as the cut-point of PD-L1 (+) and PD-L1 (–). In detail, 222 patients were identified as PD-L1 (+). In the PD-L1 CPS ≥ 10 arm, pembrolizumab showed superiority to the chemotherapy, especially in the mOS (9.3 vs. 6.7 months, HR = 0.69, *P* = 0.0074) and 12-month OS rate (43 vs. 20%). Of note, a significantly higher OSR was also seen in the pembrolizumab group than in the chemotherapy group, nearly 3-fold (21.5 vs. 6.1%, *P* = 0.006). Again, the efficacy of pembrolizumab in advanced EC with PD-L1 CPS ≥ 10 has been confirmed. Perhaps, it is feasible to set the cut-off value of PD-L1 expression at 10.

Furthermore, at the 2020 ESMO meetings, the interim analysis results of the KEYNOTE 590 trial (NCT03189719), a multi-center, randomized, double-blind, phase III study designed by Professor Kato et al. (8) were reported. It was the first time to determine the feasibility of pembrolizumab combined with chemotherapy (paclitaxel+5-fluorouracil) as first-line treatment in unresectable or metastatic EC (8). Specifically, the mOS in the general population was 12.4 months in the pembrolizumab+chemotherapy group and 9.8 months in the chemotherapy group, respectively. Among the population with PD-L1 CPS ≥ 10, the OS benefits apparently increased; the mOS was improved in the combinational therapy group compared with the chemotherapy monotherapy group (13.5 vs. 9.4 months, HR = 0.62, *p* < 0.0001), as did the 12-month OS rate (54 vs. 37%). Also, the addition of pembrolizumab reduced the risk of death by 38%.

Overall, in the treatment of EC, the population harboring PD-L1 CPS ≥ 10 definitely benefitted when pembrolizumab was given as the second-line/third-line monotherapy or as the first-line combination treatment.

*In gastric cancer*. Besides, the clinical application of pembrolizumab in GC has also encouraged researchers to further explore its value. In the cohort 1 of KEYNOTE 059 trial (NCT02335411), in the progression free survival (PFS), both the mOS and the ORRs were higher in the patients who were PD-L1 (+) than in the patients who were PD-L1 (–) when taken as third-line treatment (37). In the KEYNOTE061 trial (NCT02370498), a multi-center and phase III study explored the efficacy of pembrolizumab monotherapy for advanced patients with GC (38). Although the final analysis showed that pembrolizumab failed to significantly show differences in prolonging mOS when investigating those with PD-L1 positivity, it could improve survival in patients with PD-L1 CPS ≥ 10 after subgroup analysis. Another multi-center, randomized and positive-controlled, phase III clinical trial recruited patients with GC/EGJC identified as PD-L1 (+) who were previously untreated in order to examine the efficacy of pembrolizumab as monotherapy or in combination with chemotherapy (39). Among the PD-L1 CPS ≥ 1, the established non-inferiority endpoints were met when pembrolizumab was administered alone. Of note, further stratified analysis demonstrated that, especially in those with PD-L1 CPS ≥ 10, a remarkably longer mOS was observed in the pembrolizumab arm than the placebo+chemotherapy arm (17.4 vs. 10.8 months). Nevertheless, despite the PD-L1 CPS ≥ 1 or PD-L1 CPS ≥ 10, the regimen of pembrolizumab plus chemotherapy regimen was not superior to chemotherapy alone. In 2020, the ASCO meetings updated data from the Asian subgroup of the KEYNOTE 062 study (40). Subsequent analyses indicated that these patients had a longer mOS and higher 12- and 24-month OS rates for both PD-L1 CPS ≥ 1 and PD-L1 CPS ≥ 10. Moreover, Asian patients might have better responses and may benefit more compared with the Western patients.

Indeed, for EC and GC, PD-L1 expression has great potential to be a valid biomarker for immunotherapy, but current explorations are small-sample researches; and the cut-off value of PD-L1 CPS scoring requires further discussion. CPS = 10, as a balance between PD-L1-positive and PD-L1-negative, seems reasonable from the emerging clinical data.

##### In Hepatocellular Carcinoma

The treatment landscape for HCC has been revolutionized by the advent of ICIs. In 2018, Zhu et al. published an open-label, non-randomized, phase II trial (KEYNOTE 224, NCT02702414) assessing the efficacy of pembrolizumab in previously treated patients with HCC (41). Preliminary results showed a potential relation between PD-L1 expression and clinical response to anti-PD-1 therapy. In detail, when using CPS scoring to determine the PD-L1 status described in this study, the investigator analyzed the subset with PD-L1 (+) (*n* = 52). A strong correlation between the PD-L1 expression and the ORR and the PFS was presented. However, this subset was a small-sample and also insufficient to support the abovementioned perspective, and an expanded sample is needed for validation and reconciliation. Then, in 2019, another phase III study, KEYNOTE 240 (NCT02702401), also highlighted this association (42). Of note, pembrolizumab appears to be beneficial as a second-line option in patients with HCC who were identified as PD-L1-positive.

#### Nivolumab

Mechanically, nivolumab (another immune checkpoint blockade) is supposed to behave similarly to pembrolizumab in GC or HCC, underlying that nivolumab could also reinvigorate the T-cell function by inhibiting the PD-1/PD-L1 signaling pathway and then target TCs; however, it is not true in the real-world studies. Sometimes, regardless of the PD-L1 status, nivolumab remains effective, unlike pembrolizumab.

One representative clinical trial in EC is ATTRACTION-3 (NCT02569242), a multi-center, open-label, randomized, phase III study which was investigated to evaluate the feasibility of nivolumab for late-stage patients with ESCC who previously received treatments (4). A total of 419 patients were randomly divided into the nivolumab arm (*n* = 210) or the chemotherapy arm (*n* = 209). The PD-L1 staining was detected by IHC 28-8 pharmDx assay; patients were then stratified into subsets (PD-L1 < /≥1%, PD-L1 < /≥5%, and PD-L1 < /≥10%) based on the results of the assay to assess the interaction. Nivolumab indeed prolonged the mOS compared with chemotherapy alone (10.5 vs. 8.0 months); but intriguingly, no significant difference was observed when comparing the mOS in the subset with PD-L1 <1% with that in the subset with PD-L1 at least 1% in the nivolumab group (10.9 months, 95% CI 8.4–13.9; 10.9 months, 95% CI 8.0–14.2).

Focusing on nivolumab in GC, Kang et al. (43) launched the ATTRACTION-2 trial (ONO-4538-12, NCT02267343) involving nivolumab as the salvage therapy after standard chemotherapy for patients with GC in 2017. Among those patients receiving nivolumab, the exploratory data showed that the mOS in the PD-L1 (+) subgroup and the PD-L1 (–) subgroup were 5.22 months (95% CI, 2.79–9.36) and 6.05 months (95% CI, 4.83–8.54), respectively. The response to nivolumab in GC appeared to be independent of PD-L1 status. Similarly, at the 2020 ESMO meetings, Professor Moehler et al. (5) updated the latest data from a randomized, phase III trial of CheckMate 649 (NCT02872116). The study enrolled previously untreated patients with GC/EGJC, with or without PD-L1 expression. Approximately 60% of the participants (955/1581) were performed as PD-L1 CPS ≥ 5. Among those with CPS at least 5, comparing with the chemotherapy group (482/955), the mOS was improved in the nivolumab+chemotherapy group (473/955) (14.4 vs. 11.1 months, HR = 0.71, *p* < 0.0001), as well as the PFS (7.7 vs. 6.1 months, HR = 0.68, *p* < 0.0001). Also, among those with CPS at least 1 (1,296/1,581), the nivolumab-based combinational therapy arm (641/1,296) had a prolonging mOS (14.0 vs. 11.3 months, HR = 0.77, *P* = 0.0001) than the chemotherapy arm (655/1,296). Again, the effect of nivolumab in GC with PD-L1 (+) was confirmed.

When referring to nivolumab in HCC, the CheckMate 040 trial which involved those unselected patients with HCC further found that the response rate might not correlate well with PD-L1 expression (44). During the dose-expansion phase, the investigators stratified the available patients (*n* = 174) into the PD-L1 TPS ≥ 1% (34/174) and TPS <1% (140/174). The OSRs in the two subsets were 26 and 19%, respectively.

#### Combinational Treatment

Intriguingly, just like other solid tumors, low response rates to ICIs monotherapy (such as pembrolizumab or nivolumab alone) are common for HCC, with OSRs of ~17 and 18.7% from the most recent follow-up data from the CheckMate 040 study (NCT01658878) at the 2021 ASCO meeting, the OSR for those treated with the dual-biologic regimen of nivolumab plus ipilimumab yet reached 33% (45). Likewise, the results described in the KEYNOTE 224 trial showed an OSR of only 17% (41). Hence, the combination therapies including different ICIs, or ICIs plus targeted agents, or ICIs combined with surgery/radiofrequency ablation (RFA)/transcatheter arterial chemoembolization (TACE), opened a new horizon in HCC.

Building on the initial efforts of the IMbrave 150 trial (NCT03434379), the combinational regimen of atezolizumab (an anti-PD-L1 antibody) plus bevacizumab [a monoclonal antibody against vascular endothelial growth factor (VEGF)] has been approved as the first-line treatment in HCC (46). The literature reported that anti-VEGF might excessively cause a vascular pruning effect which would aggravate hypoxia and acidosis in the tumor tissue, and then the immunosuppression status including the up-expression of PD-L1 occurs in HCC. Angiogenesis inhibition and relieving immune suppression in tumor microenvironment (TME) are both helpful. That is why blocking VEGF and PD-1/PD-L1 signaling pathway is practicable. Similarly, in another study (named ORIENT-32) involving sintilimab (an anti-PD-1 antibody) in addition to bevacizumab, validated the feasibility of anti-PD-1/PD-L1 plus anti-VEGF agents (47). Besides, the ongoing trials, such as the IMbrave 050 study (NCT04102098), are exploring the efficacy of the above combinational protocol in patients with HCC after hepatectomy or ablation (48).

Also, scholars have combined ICI with locoregional therapies with the expectation of improving or even reversing the barriers of single-agent ICI in HCC. Similar investigations, such as NCT03397654 (a phase I/II study involving combination of TACE or RFA and pembrolizumab), IMMUTACE (NCT03572582, a phase II trial on drug-eluting bead TACE plus nivolumab), and NCT03143270, are ongoing and have indicated potential (49, 50). It can be seen that ICIs-based combination therapy is indeed superior to ICI alone in HCC, but the relevant basis remains poorly understood and more efforts are needed.

Just based on the existing results, there is still reluctance to utilize PD-L1 as the biomarker for routine therapeutic guidance of nivolumab in digestive tumors. Also, it remains controversial why some PD-L1-expressing tumors are sensitive to ICIs and others are not, and why anti-PD-1/PD-L1 responses do not differ much between PD-L1-high and PD-L1-low in some tumors. At the same time, no denying that differences in response to anti-PD-1/PD-L1 therapy are evident when receiving different PD-1 inhibitors; in addition, the diversity of evaluation approaches (including the commercial platforms and testing methods) and the subjectivity of pathologists (including the interpretation judgments) are also critical variables, which further lead to disagreements. Another term is what is the optimal threshold value of PD-L1 expression; in fact, the current trials almost choose CPS 5 or 10 as the balance, but have failed to cover the various tumor types; and sometimes, CPS 1 is used as the cut-point as well (as shown in Table 1). Future studies should focus more on the quantification of PD-L1 positivity.

**Table 1:** The clinical trials when PD-L1 expression as a biomarker.

**Trial**	**Phase**	**Treatment and target**	**Tumor type**	**PD-L1 stratification**	**Testing method**	**Endpoint**	**References**
KEYNOTE 180 (NCT02559687)	Phase II, open-label, single-arm	Pembrolizumab, PD-1	Advanced ESCC/GEJC	PD-L1 (+): CPS ≥ 10 PD-L1 (–): CPS <10	IHC 22C3 pharmDx assay	mOS, OSR, DCR	(35)
KEYNOTE 181 (NCT02564263)	Phase III, open-label, RCT	Pembrolizumab, PD-1	Advanced/Metastatic EC	PD-L1 (+): CPS ≥ 10 PD-L1 (–): CPS <10	IHC 22C3 pharmDx assay	mOS, PFS	(36)
KEYNOTE 590 (NCT03189719)	Phase III, placebo-controlled, RCT	Pembrolizumab, PD-1	Advanced/Metastatic EC	PD-L1 (+): CPS ≥ 10 PD-L1 (–): CPS <10	IHC 22C3 pharmDx assay	mOS	(8)
ATTRACTION-3 (NCT02569242)	Phase III, open-label, RCT	Nivolumab, PD-1	Unresectable/Recurrent ESCC	PD-L1 TPS ≥1, 5, 10%	IHC 28-8 pharmDx assay	mOS	(4)
CheckMate 649 (NCT02872116)	Phase III, open-label, RCT	Nivolumab, PD-1	Advanced GEJC/GC	PD-L1 (+): CPS ≥ 5 PD-L1 (–): CPS <5	IHC 28-8 pharmDx assay	mOS, PFS	(5)
KEYNOTE 062 (NCT02494583)	Phase III, RCT	Pembrolizumab, PD-1	Advanced GC/GEJC	PD-L1 CPS ≥ 1, 5, 10	IHC 22C3 pharmDx assay	mOS, PFS	(39)

*RCT, randomized clinical trial; PD-1, programmed death-1; PD-L1, programmed death-ligand 1; GC, gastric cancer; GEJC, gastroesophageal junction cancer; ESCC, esophageal squamous cell cancer; CPS, combined positive score; TPS, tumor proportion score; IHC, immunohistochemistry; mOS, median overall survival; PFS, progression free survival*.

## Tumor Mutational Burden as a Biomarker

Recently, TMB has evolved as an emerging biomarker in immunotherapy, especially in terms of drug-response and prognosis prediction (51, 52). In fact, in several cancer types, such as NSCLC, melanoma and urothelial cancer (UC), the level of TMB has been noted to be related with the clinical outcomes; for example, the higher expression of TMB after anti-PD-1/PD-L1 or anti-CTLA-4 treatment, perhaps present better clinical performance in terms of OSRs, than lower levels of TMB (10, 53, 54). In 2019, a pooled analysis involving over 100,000 tumor patients convincingly revealed a positive related correlation between TMB-high and better survival when received immunotherapy. And in contrast to chemotherapy, immunotherapy is recommended for those tumors harboring TMB-high (53). In the KEYNOTE 158 trial which involved multiple tumor types, pembrolizumab monotherapy resulted in an OSR of 29% in those with TMB-high (defined as TMB with at least 10 mutations/Mb), thereby promoting the approval of pembrolizumab alone for unresectable or metastatic solid tumors with TMB-high (≥10 mut/Mb) by FDA (55).

As an independent molecule of PD-L1, TMB also suffers from some inherent defects just like PD-L1, from the sampling stage, the detection process to the reporting stage. Hence, interfered by multiple factors, TMB sometimes fails to adequately capture which patients would likely benefit from ICIs or unlikely benefit and does not fully mirror the clinical outcomes as well.

### The Definition of TMB and Its Role

Typically, TMB refers to the number of non-synonymous mutations in somatic cells, which are usually expressed as mutations per Mb (mut/Mb). Given that cancer development can be attributed to a consistent accumulation of somatic genetic mutations, which mostly involved point, synonymous, and missense alterations, the neoantigens derived from TCs could be presented to T cells *via* the DCs after hydrolysis (56); in turn, these stimulated T cells would immediately transform into mature, activated T cells to recognize neoantigen-specific proteins as well as to increase the activated T cells; simultaneously, a minority of those neoantigens could be processed and then be placed onto the class I or II major histocompatibility complex (MHC 1 or MHC 2) which are easily discriminated and further attacked by the immune system (31, 57, 58). This is the process of recognizing “self” and “non-self” in the immune system (as shown in Figure 1). It is reasonable to conclude that the quantification of neoantigens is positively related with that of the somatic alterations (59). Likewise, the hypothesis that tumors with higher TMB level would render a better response to ICI agents is also convincing. In addition, the level of TMB could represent the mutational loads and thus estimate the neoantigens. Therefore, TMB may become a promising biomarker when acting in the response to immunotherapy (60). Another interpretation is that the deficiency of mismatch repair (MMR) genes is likely correlated with TMB-high, which also contributes to more benefit from anti-PD-1/PD-L1/CTLA-4 therapies (10). Of note, studies have suggested that several gene variations, such as TP53-related and APOBEC-related mutagenesis, might be responsible for the high-expression TMB (61, 62). And it is well-known that most TMB-high tumors occur in those with mutations in DNA POLE exonuclease domain and polymerase delta 1 (POLD-1) (63, 64). Hence, the abovementioned factors may interfere with the TMB level.

On the basis of the tissue sample, the tissue TMB (tTMB) is recognized as an accurate molecule as an approximation of tumor load, and the blood TMB (bTMB) could be a substitute for tTMB if necessary (65). Compared to tTMB, bTMB has easier access and less affected by biopsy. Analyzing the POPLAR trial (NCT01903993) and the OAK trial (NCT02008227), a positive-associated correlation between tTMB and bTMB is observed (65). More importantly, the accuracy of TMB detection, neither tTMB nor bTMB, requires to be further improved and harmonized as well.

### The Testing Methods and Interpretation of TMB

Whole-exome sequencing (WES) (300–400 gene panel in particular) is considered the gold standard for TMB assessment, but may be constrained by its high cost and judgment criteria (66). In 2016, with the emergence of commercial next-generation sequencing (NGS) detection panels called FoundationOne CDx (F1CDx) and Memorial Sloan Kettering-Integrated Mutation Profiling of Actionable Cancer Targets (MSK-IMPACT), they have been approved as the accompanying diagnostic products in pan-tumor types (67, 68). Multiple testing approaches are available in the clinical setting. In principle, the difference between WES and NGS large panels only lies in the size of the area covered by the probe. In addition, the accuracy of panel-based TMB is strongly affected by the corresponding panel size, since it has been shown that TMB calculations are more precise in panels with gene sizes between 1.5 and 3.0 Mb; conversely, for those with smaller-size panel, the TMB quantification might be less reasonable (69). Similarly, Budczies et al. (70) also described such a confident correlation between TMB and the panel size. In detail, sensitivity and specificity increased but uncertainty decreased as gene size expanded; in particular, the inaccuracy of the TMB increased for sizes smaller than 1 Mb. Also, the sampling quality can confound the TMB level; multi-region biopsy and high-quality formalin-fixed paraffin-embedded (FFPE) sample are recommended. Another factor that affects TMB measurement is its corresponding calculation method. Taking the WES–TMB assessment as an example, it only involves missense variations (71). In contrast, the MSK-IMPACT panel-based TMB method always takes non-synonymous alterations into account (67, 71); yet, the F1CDx counts not only synonymous mutations, but also non-synonymous mutations (71).

Meanwhile, no general consensus has been reached on the definition of TMB-high and TMB-low. And the common approach in clinical studies is based on the statistics percentile stratification, while some adopt the receiver operator characteristic (ROC) curve analysis, which seems to be a more reliable method. TMB generally ranges from 100 to 248 mutationgenesis, or from 5 to 37 mut/Mb across tumor types *via* various detection approaches. Taking the TMB in CRC as an example; in 2018, to identify the optimal-responding sub-population after anti-PD-1/PD-L1 therapy, a percentile was used to define TMB-high as ≥12 mutations/Mb (72). In another retrospective trial, the designers set the cut-off value for TMB-high and TMB-low as 37 mut/Mb by log-rank statistics (73). However, the cut-off value of 10 mut/Mb is regarded more valid and appropriate in NSCLC according to previous trials (such as the CheckMate 568 study) (74). The above findings also suggest that it may not be feasible to define TMB-high and TMB-low with a generic threshold in immunotherapy for multiple cancer types; instead, targeting of the specific tumors and then setting the corresponding cut-off values seem to be more persuasive than received immune checkpoint inhibitions.

### The Clinical Utility of TMB

Tumor mutational burden is extensively utilized in NSCLC and melanoma as a predictive tool for checkpoint blockade-based immunotherapy, but few researches have emphasized digestive-system cancers (75, 76). Kim et al. (77) analyzed 55 patients with GC after PD-1 inhibitions and pointed out that the level of TMB was correlated with the efficacy of immunotherapy. In CRC, particularly in those CRC harboring microsatellite instability high (MSI-H) or deficiency of MMR (dMMR), TMB could be better captured in those who were ineffective with immunotherapy through an exploratory study in 2019 (73) (Table 2). In general, TMB seems to be a valid predictor of response to ICIs-based immunotherapy for EC, GC, and CRC, but its status remains further confirmed as the current trials are almost small samples, and there are no large-scale and phase III clinical studies to verify them.

**Table 2:** The clinical trials when TMB as a biomarker.

**Trial**	**Phase**	**Treatment and target**	**Tumor type**	**TMB stratification**	**Testing method**	**Endpoint**	**References**
NCT02915432	Phase Ib/II, multi-center	Toripalimab, PD-1	Advanced GC/ESCC	TMB-high ≥ 12 mut/Mb TMB-low <12 mut/Mb	WES	mOS	(78)
KEYNOTE 061 (NCT02370498)	Phase III, multi-center	Pembrolizumab, PD-1	Advanced GC	TMB-high > 175	MSK-IMPACT	–	(9)
NCT02870920	Phase II, RCT	Durvalumab, PD-L1	Recurrent CRC	TMB-high ≥ 28 mut/Mb TMB-low <28 mut/Mb	F1CDx	mOS	(79)
REGONIVO (NCT03406871)	Phase Ib, open-label	Nivolumab, PD-1	Advanced GC/CRC	TMB-high ≥ 22.55 mut/Mb TMB-low <22.55 mut/Mb	NR	mOS	(80)
Morris et al. (12)	–	ICIs	Multiple tumor types	CRC: TMB-high ≥ 52.2 mut/Mb EC: TMB-high ≥ 8.8 mut/Mb	MSK-IMPACT	–	(12)

*RCT, randomized clinical trial; PD-1, programmed death-1; PD-L1, programmed death-ligand 1; GC, gastric cancer; EC, esophageal cancer; ESCC, esophageal squamous cell cancer; CRC, colorectal cancer; TMB, tumor mutational load; mOS, median overall survival; WES, whole-exome sequencing; MSK-IMPACT, Memorial Sloan Kettering-Integrated Mutation Profiling of Actionable Cancer Targets; F1CDx, FoundationOne CDx*.

#### In Gastric Cancer

In 2018, Kim et al. (77) led a phase II trial which recruited 55 patients with GC who were under pembrolizumab monotherapy. The OSRs in the TMB-high subset [at least 400 non-synonymous single nucleotide variants (SNVs)], the TMB-moderate subset (100–400 SNVs), and the TMB-low subset (<100 SNVs) were 88.9, 20.0, and 11.1%, respectively. This is the first time to reveal the potential correlation between TMB level and clinical response in GC. Subsequently, in another multi-center and phase Ib/II clinical study (NCT02915432) conducted by professor Ruihua Xu, toripalimab monoclonal antibody (an anti-PD-1 agent) was used for advanced GC and ESCC (78). Specifically, they defined TMB-high as ≥12 mut/Mb, while TMB-low as <12 mut/Mb. The improved effective rate (33.3 vs. 7.1%, *P* = 0.017) and mOS (14.6 vs. 4.0 months, HR = 0.48, *P* = 0.038) were observed in the TMB-high arm than the TMB-low arm. Indeed, TMB shows promise as a biomarker in immunotherapy for GC. In 2019, scholars assessed 161 patients with EGJC who were treated with anti-PD-1/PD-L1/CTLA-4 agents (11). TMB was estimated *via* MSK-IMPACT sequencing. Interestingly, an increase in TMB was associated with longer survival; this correlation yet disappeared in multi-variable analysis when those patients with MSI-H were excluded. Given the fact that the MSI subtype in GC has high levels of TMB and a better response to immune checkpoint inhibitions (10), it suggested that this improvement in outcome may be a result of those patients with MSI-H.

The 2020 ASCO meetings reported on the KEYNOTE 061 trial, which investigated the relationship between TMB and PD-L1 expression (81). Among those TMB-high participants (which was defined as TMB > 175), pembrolizumab was superior to paclitaxel; besides, no significant correlation was seen between TMB and PD-L1, demonstrating that TMB could serve as a PD-L1-independent biomarker. In addition, the role of TMB in predicting the efficacy of ICIs-based therapy was also supported by Shitara et al. (82) who profiled TMB through the F1CDx panel. In brief, the value of TMB in GC immunotherapy cannot be ignored and need further attention.

#### In Esophageal Cancer and Colorectal Cancer

Comprehensively analyzing 1,662 patients with multiple tumor types undergoing ICI (including 110 patients with CRC and 26 patients with EC), Prof. Morris et al. found that a higher tumor mutational load always accompanied a better overall outcome (12). Whereas, the cut-off value of TMB in various malignancies could be distinct across cancer types with regard to being predictive of immunotherapy; for example, a TMB cut-point of 52.2 mut/Mb was considered to be feasible in CRC, while that for EC was 8.8 mut/Mb.

In 2019, 22 metastatic patients with CRC carrying MSI-H who had received anti-PD-1/PD-L1 blockades were enrolled (73). According to response evaluation criteria in solid tumors (RECIST) standards, seven patients achieved a complete response (CR), eight patients achieved a partial response (PR), one patient achieved stable disease (SD), and six patients had disease progression (PD), respectively. The OSR reached ~68%. The TMB-analysis showed that the median value of TMB in those who had CR and PR was obviously higher than that in those who had SD and PD; simultaneously, the optimal cut-point for TMB ranged from 37 to 41 mut/Mb. At the ASCO-GI 2019 meetings, the CCTG CO.26 trial (NCT02870920) compared the efficacy of the combinational regimen including a PD-L1 inhibitor durvalumab plus a CTLA-4 blockade tremelimumab with best supportive care (BSC) alone for advanced CRC (79). The investigators set the cut-point of TMB as 28 (TMB-high: TMB ≥ 28 mut/Mb; TMB-low: TMB <28 mut/Mb). Among those with TMB <28 mut/Mb, no significant survival benefits were observed in the combination group; however, compared with the BSC group, the doublet-ICI regimen significantly improved the mOS (5.5 vs. 3.0 months, HR = 0.34). Then, taking the focus into the REGONIVO study (NCT03406871), the presetting cut-off value of TMB was defined as 22.55 mut/Mb (80). The included patients with CRC were divided into TMB-high arm and TMB-low arm. The OS of TMB-high subgroup was longer than that of the TMB-low subgroup (12.5 vs. 7.9 months). Undeniably, TMB helps to select better-responders in MSI-H-type CRC, as well as to distinguish potential-candidates for microsatellite-stable-type (MSS-type) CRC.

Considering the encouraging results and strong evidence of TMB in GC and CRC, we could assume that TMB is also a promising biomarker with independency of PD-L1. Yet, several issues exist in TMB detection. Firstly, we must acknowledge that not all mutations could be as neoantigens, which is actually a huge obstacle in differentiating neoantigen and further predicting the corresponding effects. Other factors, such as different TMB algorithms for WES-based and NGS-panel-based methods, the quantitative disagreements including variant types, variant allele frequency (VAF), and cut point, and the inconsistencies during reporting, also interfere with the accuracy of TMB assessment. More significantly, so many variables could lead to TMB-high, for example, MSI-H, gene variations (TP53, RRM1, FANCE, and POLG, etc.). Notably, the patients with POLE exonuclease domain mutations (EDMs) are thought to have a better prognosis (83). And in another pan-tumor analysis, the scholars counted the prevalence of POLE/POLD variations among 47,721 cancer patients, 2.79 and 1.37%, respectively; and the TMB level in those with such mutations was obviously higher than those without mutations (84). The OS showed a similar trend, that in the patients with POLE/POLD-mutated, the OS was superior to that in the non-mutated patients (34.0 vs. 18.0 months, *P* = 0.004). Multi-variable analysis indicated that POLE/POLD variation might be a novel and alternative biomarker in immunotherapy-based treatments and also independent of MSI-H as well. Hence, it is equally crucial to balance various biomarkers based on TMB when detecting TMB levels to reduce the associated effects and further improve the accuracy. At the same time, the corresponding cut-off value to define TMB-high and TMB-low in diverse cancer types shows a significant difference; and perhaps, determining a specific cut-point for a particular tumor is essential.

## Microsatellite Instability Status as a Biomarker

Microsatellite instability-high or deficiency of MMR (dMMR) frequently occurs in multiple cancer types, such as endometrial carcinoma, followed by CRC, GC, and HCC. Evidence has suggested that MSI-H-type CRC (especially referring to stage II) and endometrial carcinoma have a better prognosis than MSS-type tumors but its role in other cancers is still not clear (10). With the rapid approval of pembrolizumab as the first-line treatment for metastatic CRC with MSI-H or dMMR in June 2020, the status of MSI is increasingly elevating.

### The Definition of MSI-H/dMMR and Its Role

Mismatch repair genes mainly comprise MLH 1, MSH 2, MSH 6, and PMS 2, and alterations, which would cause the loss of MMR, representing dMMR, the deletion or variation of individual gene fragments, which then consistently accumulate DNA errors and are passed on to the next generation, with progressive malignant transformation. As the mutated base pair fragments are extended, the more repetitive the base sequences, the higher the instability of microsatellite. This is known as MSI-H. Specifically, the MLH 1 methylation [also called CpG island methylator phenotype (CIMP)], the epigenetic inactivation of MSH 2 and the downregulation MMR genes mediated by mRNAs may produce MSI status. Besides, previous findings have elucidated that a higher level of cytotoxic cells, such as TILs, would be frequently seen in the MSI-positive tumors, which in turn perform greater immunogenicity and thus induce better immunotherapeutic responses.

In fact, MSI is normally classified into three subtypes, MSI-H, MSI-low (MSI-L), and MSS; moreover, different subtypes also own their unique clinicopathological features. For example, the presence of MSI-H typically represents a good outcome in GC. At the ASCO meeting in 2019, a large meta-analysis involving four randomized clinical trials (the MAGIC, CLASSIC, ARTIST, and ITACA-S studies) discussed the predictive value of MSI status in resectable GC (85). Totally, 1,552 participants were included, and ~7.8% (121/1552) of whom were diagnosed with MSI. Compared with the MSS-type subgroup, the 5-year OSR (77.4 vs. 59.2%, HR = 0.50, *p* < .001) as well as the 5-year disease-free survival (DFS) (71.8 vs. 52.3%, HR = 0.50, *p* <0.001) in the MSI-type subgroup was better. Furthermore, MSI was independently associated with the DFS (HR = 0.48, 95% CI 0.33–0.70, *p* <0.001) and the OS (HR = 0.48, 95% CI 0.29–0.81, *P* = 0.005). Thus, MSI is indeed an independent factor when predicting a good prognosis and a specific element in patient stratification.

### The Testing Methods and Interpretation of MSI-H/dMMR

The conventional approaches have used protein-level-based IHC techniques that rely on testing the expression of four MMR proteins or molecule-level-detection PCR, which directly measures changes in the mononuclear or binuclear glycoside repeat sequences. By comparison, the results from the two methods remain consistent in most cases, but occasionally inconsistent and some even the opposite. In 2019, a single-center, prospective trial that included 38 patients with a diagnosis of MSI/dMMR (+) mCRC from the CheckMate 142 and KEYNOTE 164 study demonstrated that misjudgments might exist as a result of traditional detection methods (referring to the PCR-based and IHC-based technique) after re-verification and partially caused primary drug-resistance to immune checkpoint inhibitions (86). In detail, among five patients with primary resistance, the MMR testing and MSI status were misjudged in ~60% (3/5) of them. Furthermore, analyzing another 93 patients, the results showed that ~10% (9/93) had false-positive MSI, among whom the discrepancy between the IHC and the PCR reporting was observed in three patients. Therefore, detecting both MSI status and MMR proteins prior to ICIs is recommended, and this undoubtedly requires an increased sample size, which would increase the detection cost as well.

Specifically, from IHC analysis, MSI is defined as the absence of any MMR genes (MLH 1, PMS 2, MSH 2, and MSH 6), otherwise referred to as MSS; moreover, if one MMR gene deletion exists, it is described as MSI-L, and if at least two gene deletions are present, it is defined as MSI-H. Overall, IHC is characterized by easy operation and low-cost, but it cannot identify whether dMMR is derived from other genes other than the four MMR genes, which in turn affects the application of ICIs.

As for PCR, multiplex fluorescent PCR combined with capillary electrophoresis (CE) is widely utilized in current trials (such as the CheckMate 142 study) to determine MSI status *via* PCR detection of specific microsatellite repeats while comparing *in situ* mutations in tumor specimens with matched normal tissues. Notably, five microsatellite loci (BAT25, BAT26, D5S346, D2S123, and D17S250 included) are recommended by the National Cancer Institute (NCI) based on the Bethesda guideline (87); of them, the germline alterations occur in more than two sites and identified as MSI-H, where only one site is MSI-L and no site is determined as MSS. The marker of BAT25 and BAT26 belongs to mononucleotide molecule, while D5S346, D2S123, and D17S250 are dinucleotide markers which have a lower sensitivity and specificity than the mononucleotides in subsequent researches. Then, Bacher et al. (88) analyzed 266 microsatellite markers, ranging from mononucleotide to pentanucleotide molecule, and found that the mononucleotides were the markers with the best sensitivity and specificity. Furthermore, the Promega analysis system was proposed, which contains five single-nucleotide microsatellite loci (BAT25, BAT26, NR21, NR24, and MONO27) in addition to two pentanucleotides (Penta C and Penta D) to detect MSI, which is relatively less time-consuming and expensive. Strictly speaking, which is the most available microsatellite marker for PCR detection is still controversial, since various panels included multiple sites on the market. Another factor is the racial differences between the East and West. For example, for East Asian population, PCR *via* NCI panel is more applicable, which was confirmed in 2018 (89). And a follow-up research in 2019 equally supported this idea (90). In spite of PCR being the gold criteria when detecting MSI status, it is limited by its complicated process when matching samples and the potential judgment bias.

At present, another approach, the NGS-panel-based MSI assessment, simultaneously sequences millions of gene molecules. Evidence in a large-sample study suggested that MSI assessment *via* NGS had better sensitivity than the PCR-based method (91). In 2019, Trabucco et al. (92) introduced a novel NGS-based MSI identification designed by FoundationOne CDx that analyzed 67,644 patients' FFPE samples. Remarkably, this approach was extremely reliable as it achieved ~97% consistency with IHC-based or PCR-based methods. Besides, it need not match to the normal tissues and could detect both MSI and TMB, thus it reduced the demand for specimens. Actually, the overall prevalence of MSI in over 1,000 tumor samples varied by tumor types; some cancers were with higher MSI positive rate, such as endometrial carcinoma (16.5%), small intestinal carcinoma (4.6%), CRC (4.5%), appendiceal carcinoma (4%), and GC (3.4%). Yet, in other caner types, such as melanoma and lung cancer, MSI is rare or even absent. A un-uniformity distribution of MSI-type malignancies across cancer types is typical. Hence, this new method allows it to detect MSI in all tumor types, particularly those that cannot use the conventional PCR/IHC methods as a result of rare low incidence. More importantly, further analysis pointed out the specific signaling and gene enrichments in MSI-H (+) (including WNT, PI3K, and NOTCH pathways) and MSS (+) (including APC and CTNNB 1) tumors, which presumably guide the combination selection in therapeutic decision making.

### The Clinical Utility of MSI-H/dMMR

#### In Colorectal Cancer

Actually, the MSI status is a widely-applied biomarker in CRC treatments, since the MSI-H/dMMR subtype CRC has its unique features; furthermore, its response to ICIs (+) is evident.

In 2015, a phase II clinical trial, named KEYNOTE 016 (NCT01876511), was conducted to explore the value of MSI in anti-PD-1 therapy (pembrolizumab) for advanced tumors with MSI-H/dMMR positive (93). Forty-one included patients were then divided into three cohorts, the CRC with MSI-H/dMMR (+) cohort, the CRC with non-MSI-H (+) cohort, and the non-CRC tumors with MSI-H (+) cohort, respectively. The OSRs in three such cohorts were 40, 0, and 71%, and the 20-week PFS rate was 78, 11, and 67%. The encouraging data from the KEYNOTE 016 study served as a catalyst for the rapid approval of pembrolizumab in those solid tumors harboring MSI-H/dMMR by the FDA, and also in advanced CRC after the failure of conventional chemotherapies. Also, it appeared that the effect of pembrolizumab was durable for metastatic CRC with MSI-H/dMMR (+), as well as the manageable toxicities which have been confirmed in another multi-center, phase II trial (KEYNOTE 164, NCT02460198) (94). Then, taking the focus to the KEYNOTE 177 study (NCT02563002) presented in the 2020 ASCO meetings (95). There were 307 CRC candidates diagnosed with MSI-H/dMMR positivity who were randomized into the pembrolizumab monotherapy arm (*n* = 153) or the chemotherapy arm (*n* = 154) (95). The results elucidated that the immune-related PFS was longer than the chemotherapy-related PFS (16.5 vs. 8.2 months, HR = 0.60, *P* = 0.0002), almost doubled; and a similar trend was seen in the 12-month PFS rate (55.3 vs. 37.3%) and 24-month PFS rate (48.3 vs. 18.6%). Intriguingly, the incidence of grade 3–5 in the pembrolizumab group was lower than that in the chemotherapy group (22 vs. 66%). No denying that the position of pembrolizumab in MSI-H-type CRC has been graduately established nowadays.

Subsequently, the investigators designed a multi-center, open-label, phase II study (CheckMate 142, NCT02060188) which totally enrolled 119 patients with MSI-H/dMMR (+) CRC to confirm the potential of nivolumab plus ipilimumab in 2018 (96). The OSR was 54.6% (95% CI 45.2–63.8%). Similarly, based on it, FDA also has approved nivolumab for MSI-H/dMMR (+) CRC. The ASCO meetings in 2020 offered the updated results of a 2-year follow-up in the CheckMate142 trial; the OSR reached 69%, and the CR rate was 13%, better than that in 2018 (97). In addition, the 24-month PFS rate was 74%, and the 24-month OS rate was 79%.

#### In Gastric Cancer

From the molecular characteristics of GC, the Cancer Genome Atlas (TCGA) categorized GC into four subtypes; and among them, the MSI-subtype is the major component (98). It appears that patients with MSI will favorably respond to immunotherapy. Specifically, Kim et al. (77) comprehensively discussed the features in GC when sensitive to the immune checkpoint blockades. And the data indicated that the existence of MSI-H might be a potential cause, as these patients with MSI-H had an OSR of almost 87.5% (*n* = 7). However, its sample size was not enough to interpret the phenomenon. Besides, in the cohort 1 of the KEYNOTE 059 trial (NCT02335411), when considering the role of MSI status in anti-PD-1treatment, the OSR of those MSI tumors (*n* = 7) was significantly higher than that of those non-MSI tumors (*n* = 167) (57 vs. 9%) (37). In 2019, the subgroup analysis of the KEYNOTE 062 study demonstrated an improved clinical outcome after anti-PD-1 therapy that ignored PD-L1 expression in the MSI-H population (39).

Overall, it is obvious that the MSI status does play a predicting role in CRC and GC. The prevalence of MSI is not yet high; in other words, MSI cannot fit for most tumor types of the digestive system. For example, the incidence of MSI-H in patients with HCC is merely 2%, and the immune-related response to pembrolizumab in those patients with HCC failed to show superiority (99, 100); hence, MSI might not be an available biomarker for HCC immunotherapy. Another crucial issue is that the MSI detections, either PCR/IHC-based or NGS-based, also need to be increasingly optimized.

## Circulating Tumor DNA as a Biomarker

**Figure 3:**
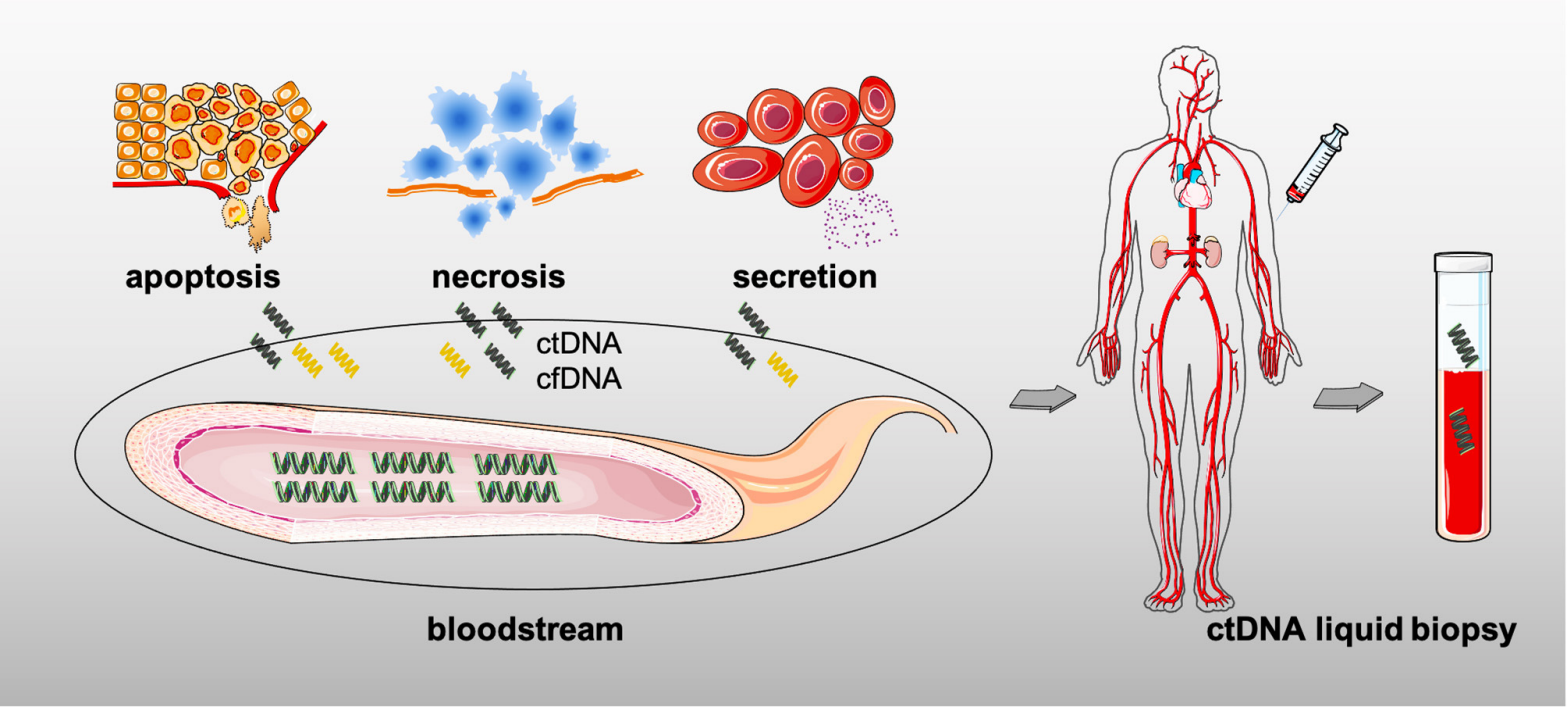
The origins of circulating tumor DNA (ctDNA).

The serum ctDNA mainly consists of genomic DNA fragments released by the TCs after apoptosis, necrosis, or active secretion, that is to say that all tumors theoretically produce ctDNA (Figure 3) (101). The information on genetic variation can be detected in ctDNA, from simple point mutation to complex structural variation, and even chromosome copy number variation (102). Critical for early diagnosis or late relapse monitoring, ctDNA from peripheral blood may better respond to intratumoral features than those invasive approaches, such as its inherent tumor-heterogeneity, the tumor loads, and the whole genetic variations, thereby serving as a pre- or post-treatment biomarker in solid tumors (103–105).

### The Definition of ctDNA and Its Role

When the term “ctDNA” is mentioned, it is a constituent of cell-free DNA (also known as cfDNA) that comprises a specific-length (normally 150–200 base pairs) and double-stranded DNA, but with a shorter fragment length and half-life as well (102, 106, 107). Numerous studies have illustrated that the hematopoietic cells could be generally the origins of cfDNA in normal tissue due to their ease of entry into the bloodstream (108); in addition, cfDNA levels increase with the emergence of malignancy, infection, inflammation, or other stress conditions (108).

The shift in the existing treatment pattern toward individualized and precision therapy in clinical practice has given the ctDNA (typically from liquid biopsy) the opportunity to participate in the anti-tumor treatments thanks to its advantages, especially compared with the conventional tissue biopsy (109, 110). First of all, detecting ctDNA is performed through non-invasive methods rather than those, such as biopsy or surgery, sometimes just by drawing some peripheral blood (as shown in Figure 3). It means that the quantity and quality of the sample is less demanding in case of insufficient sampling (111). And the short half-life also makes it convincing for dynamical monitoring of disease progression. Meanwhile, ctDNA could be attributed to multiple DNA shedding from different tissues and cells as well; then, it is not hard to understand that the serum ctDNA more comprehensively reflects the whole tumor information due to the accessible decoding of the tumor spatial and temporal heterogeneity, whereas biopsy partially represents part of the tumor it obtains (the primary or metastatic lesions) (111). Furthermore, just as mentioned in the earlier findings, ctDNA assessment facilitates early-screening and recurrence discovery, unlike the tissue biopsy which could only be further detected after a rapidly growing lesion. Hence, ctDNA assessment is non-invasiveness and dynamic with real-time monitoring and comprehensive insight along with another key point, early-prediction (112). In addition, the role of blood sample by liquid biopsy has to be mentioned when considering the acquired drug-resistance, such as the detection of epidermal growth factor receptor (EGFR) mutations in lung cancer (113); moreover, it is believed that molecular resistance is seen earlier than clinical resistance, and early finding of molecular resistance is also conductive to early-intervention. The immune-related factors including ct-DNA, circulation tumor cells (CTCs), exosomal PD-L1, T-cell receptor (TCR), and circulation RNAs (such as mRNA, microRNA, and LncRNA) in peripheral blood would greatly aid precision medicine in cancer treatments (111, 114–116). Nevertheless, entering into the clinic is still challenging for ctDNA owing to the lack of large sample trials.

### The Testing Methods of ctDNA

Initially, PCR-based techniques were characterized by low cost and convenience as ctDNA identification and quantification methods. But with the demand for higher detection sensitivity and accuracy, or as a compensation for traditional PCR approaches, droplet-based digital PCR (ddPCR) and NGS-based methods gradually emerged as the standard assessment for ctDNA-profiling. In general, the so-called first-generation sequencing methods based on PCR, including digital PCR (dPCR), ddPCR, amplification refractory mutation system (ARMS), or breads, emulsification, amplification and magnetics (BEAMing), are through detecting the predefined variations so that they are characterized by shorter turnaround times but rather relatively high sensitivity (117–119). Yet, the NGS-based approaches could cover a wider range of mutations and even entire genomes; therefore, longer turnaround times and higher prices are inevitably needed. In other respects, the corresponding sensitivity also increases when rare or novel genetic alterations as well as the epigenetic modifications are involved (Table 3).

**Table 3:** The comparison of various ctDNA detection methods.

**Type**	**Technique**	**Stability**	**Sensitivity**	**Cost**	**Target**	**Features**
ARMS-based	ARMS	Mediate	Low	Low	Single gene mutation	Convenient, easy-operation
	Super-ARMS					Higher sensitivity than ARMS
dPCR-based	ddPCR	High	High	Low	Single gene mutation	Available for the clinic
	BEAMing					Definite ctDNA-quantification
NGS-based	TAM-Seq	High (influenced by laboratory level)	High	High	Multiple gene mutations	Less cost and time, and lower sensitivity than other NGS-based methods
	Safe-SeqS					Identification of rare genetic mutations
	CAPP-Seq					“Filter”; relatively high sensitivity
	iDES					Higher sensitivity than CAPP-Seq
	TEC-Seq					Deep sequencing; potential in the early-diagnosis when no symptoms occur
Mass spectrometry	MassARRAY	–	Mediate	Mediate	Multiple gene mutations	Less range than NGS-based methods
Others	EFIRM	–	Mediate	Low	Multiple gene mutations	Short time; easy-operation

*ARMS, amplification refractory mutation system; PCR, polymerase chain reaction; ddPCR, droplet- based digital PCR; BEAMing, breads, emulsification, amplification and magnetics; TAM-Seq, tagged-amplicon sequencing; Safe-SeqS, safe sequencing system; CAPP-Seq, cancer personalized profiling by deep sequencing; iDES, integrated digital error suppression; TEC-Seq, targeted error correction sequencing; EFIRM, electric field-induced release and measurement*.

#### The ARMS-PCR-Based Testing Methods of ctDNA

The basic principle of ARMS-PCR, which was established to detect the known point mutations by Newton et al., is that the 3′-base in primer must be complementary to their template DNA for effective amplification *via* PCR technology (117). Specifically, according to given variant sites, the predesigned primers are matched to the mutant-type and the wild-type DNA templates; and then, PCR amplification is consistently performed to distinguish mutants and non-mutants (117). To ensure its accuracy and specificity, the so-called primers are in particular critical. In 2019, Lianidou et al. introduced a novel ARMS-based technique [NaME-PrO-assisted ARMS (NAPA)] to reduce the false-positive rate by inserting the enzymatic digestion step through oligonucleotide-probes (with high affinity for wild-type DNA) (120). Another Super-ARMS method also has a higher sensitivity (0.01–0.2%) than ARMS and is also presumed to be more feasible for clinical promotion. Indeed, the ARMS-PCR method is simple and convenient, but rather highly precise; nevertheless, one major constraint is its limited targeting alterations referring to the presetting mutations.

#### The dPCR-Based Testing Methods of ctDNA

In contrast to ARMS-PCR described above, dPCR adopts an absolute quantification and even counts the specific number of DNA molecules. ddPCR and BEAMing are considered to be representative assessments. To clarify the consistency between the above two methods (ddPCR and BEAMing), Nicholas et al. did a comparison in 2019 and found a good agreement in ctDNA identification, as well as excellent repeatability (121). In addition, another approach, microfluidic digital PCR, often uses a microfluidic chip.

In terms of ddPCR, the DNA isolated from plasma is divided into multiple small droplets and then placed in different micropores in order to perform PCR-induced DNA amplification by specific chemical reagents and dye probes (122). If a positive molecule (referring to ctDNA) is detected, the corresponding signal accumulation would be presented. Then, by calculating and quantifying the signal accumulation for each pore, we could obtain the level of ctDNA in the original sample with a high sensitivity of at least 0.001%. As a result, ddPCR is common in early tumor screening, but just like ARMS-PCR, it is also indispensable for those unknown variations and high-throughput sequencing.

Based on four main steps of magnetic beads, emulsion, amplification, and magnetism, another ctDNA detection method called BEAMing, which is combined with dPCR plus flow cytometry, can clone DNA *via* magnetic beads (123). Of note, the target mutant region is amplified using specific PCR primers and then mixed with magnetic beads for a water-in-oil single molecule amplification reaction. After de-emulsification, fluorescent probes of various colors fluoresce in red or green when bound to the PCR product on the magnetic beads. Subsequently, the relevant mutations are determined through the color-analysis using a flow cytometer. Obviously, BEAMing is more complex and costly.

#### The NGS-Based Testing Methods of ctDNA

Unlike the PCR-based approaches, the NGS-based techniques are characterized by high throughput, high sensitivity, and large coverage (ranging from the whole exome/the whole genome to limited genome sequencing). Currently, the targeted deep sequencing methods include tagged-amplicon sequencing (TAM-Seq), safe sequencing system (Safe-SeqS), cancer personalized profiling by deep sequencing (CAPP-Seq), and targeted error correction sequencing (TEC-Seq) as the novel and widely accepted ct-DNA detection tools to enrich the target fragments by PCR or hybridization capturing (124–126).

The core of TAM-Seq is the design of specific primers to amplify the target region two times (125). In detail, amplicons <200 base pairs (bp) in size are generated during the pre-amplification process. Then, during the labeled amplification phase, the amplified regions with mutations are selectively amplified by single-plex PCR in order to exclude non-specific products. The final results are obtained by single-end sequencing by adding a joint and a specific barcode at both ends of the amplified products. Despite the reduction in time and cost, its sensitivity needs to be improved.

Turning to Safe-SeqS, this technology first requires assigning a unique identifier to each DNA template; and the next step is to further amplify the products, which then generates numerous sub-molecules with the same sequence (124). If higher than 95% of the PCR-produced product with the same marker also contains the same variations, it means that this product is a true mutant-sequence. One of the highlights of Safe-SeqS is its ability to identify rare mutations.

Intriguingly, another technique called CAPP-Seq is known as its “filter,” which consists of bioacylated oligonucleotide probes, since it could directly target the mutation regions for ctDNA quantification (126). Of note, the sensitivity of CAPP-Seq is extremely high.

### The Clinical Utility of ctDNA

As a reliable complement to tissue biopsy, ctDNA *via* liquid biopsy has initially shown great capacity in the early-diagnosis, efficacy-assessment (especially the judgment of pseudoprogression) and prognosis-evaluation in CRC, GC, and HCC, with a minimally-invasive and convenient procedure (127–129). Scholars conducted a perspective trial involving CRC, and data suggested that the presence of ctDNA was potentially associated with the staging (130). More recently, at the 2020 ASCO meetings, a clinical study was reported that highlighted whether ctDNA could be served as a response and resistance prediction tool in patients with GC who were treated with pembrolizumab (129). Worthy to note that subsequent analysis demonstrated that the re-appearance of ctDNA during treatments, even in the earlier stage, would indicate tumor progression. Moreover, a longer PFS was also seen among those participants whose ctDNA was cleared within 9 weeks than in those who failed (12.3 vs. 3.9 months). Similarly, in 2020, the ESMO meetings presented the CALIBRATION trial (NCT03653052), which also indicated that the changes in ctDNA would predict durvalumab-related efficacy in advanced esophageal adenocarcinoma (131). But these explorations are not premature.

Indeed, ctDNA is promising in cancer treatments (mostly as a pre-chemotherapy or post-chemotherapy marker) and is not yet sufficiently credible in immunotherapy. The emergence of liquid biopsy could make up for the lack of tissue biopsy and better meet the demands of precision medicine, but its clinical popularization in the future urgently requires more large sample and well-designed trials. Meanwhile, standardization and optimization of ctDNA testing appears to be equally meaningful.

## Conclusions and Future Perspectives

An enhanced understanding of the tumor-associated immunity in turn promotes the wide application of ICIs in gastrointestinal cancer treatments over time. Distinct from the targeted therapeutic agents that generally act through those defective signaling pathways, such as the EGFR path, ICIs always fundamentally target the host immune system and then block the immune escape of TCs (29). Mechanically, an excellent and durable clinical response to immunotherapy could be undoubtedly seen in patients with cancer; but the fact is, based on the initial data from the last few decades, the response rate always reached <20% in most solid tumors, some of which may have no response or even oppositely suffer from hyperprogressive disease (HPD) or pseudoprogression. As a result, how to distinguish responders from non-responders from the whole population by specific biomarkers and how to further explore the tumor-associated immune-microenvironment landscape are vital in the era of precision therapy.

Of note, the value of PD-L1 expression in GC and EC immunotherapy-based treatments has been confirmed, while merely being less predictive in CRC. In contrast to PD-L1, evidence has shown that MSI-H/dMMR may better reflect the immune-related responses in gastrointestinal malignancies. But in HCC, it seems there is not one suitable biomarker. Strictly speaking, TMB, ctDNA, and TIL are more likely validated for pan-tumor types, rather than just a particular tumor alone. However, even when utilizing the same biomarkers, different predictive results are common across various ICIs (such as pembrolizumab and nivolumab) and distinct cancer types (such as GC and EC); in other words, these molecules are subject to their innate limitations, as well as the bioinformatic-technique and personnel errors. Actually, there are several novel and alternative biomarkers, including TIL, Epstein–Barr virus (EBV) infection, gut microbiome, POLE/POLD variation, etc. (84, 132, 133). Taking the gut microbiome as an example, its role in modulating adaptive immunity has been reported in CRC before (134). Moreover, the scholars found a high degree of discordance in primary and metastatic lesions of multiple biomarkers *via* a multidimensional analysis in 2020 (135). The discordance rate of PD-L1 (26%) and TIL (39%) between primary metastatic tumors were relatively higher than that in TMB (no significant difference) and MSI status (6%), indicating that the PD-L1expression and TIL are highly heterogeneous and likely to be disturbed by sampling sites. Overall, PD-L1 and MSI status appear promising as a predictive biomarker in immune checkpoint blockade treatments, but are indeed not ideal. A viable strategy is to combine with multiple biomarkers, such as PD-L1 plus EBV in GC and PD-L1 plus MSI-H in CRC, thereby maximizing its predictive effect in immunotherapy, and just relying on a single biomarker.

Additionally, another obstacle might be the incidence of ICIs-induced HPD, which is a common cause of reduced or even reversed efficacy during immunotherapy. As a result, it would be of interest to monitor for hyperprogression through several markers. Evidence has demonstrated that amplification of murine double minute 2/4 (MDM2/4) is associated with HPD in solid tumors (including GC and EC) (136, 137); also, EGFR mutations might be a potential cause that could drive immune-related drug-resistance, but they are less likely in digestive system tumors (138).

Advances in liquid biopsy could compensate for the traditional tissue biopsy, especially in these highly heterogeneous tumors. Scholars have proposed that a modified staging criteria implementing “B” staging into the primary “TNM” staging (defined as “TNMB” classification) would accurately represent the tumor aggressiveness, but more data are still needed (112). The so-called “B” includes ctDNA message in the blood. Exploration is reported to be in the initial phase. High cost generally comes with sensitivity. Therefore, whether liquid biopsy is ready for daily popularizing is controversial. Besides, biomarkers are strongly required and increasingly evolving in the immunotherapy landscape of digestive system malignancies, but always challenging. For example, ICIs have been successfully applied and is emerging as a mainstay in HCC; however, no available biomarkers that can play a perfect predictive role. Although ctDNA shows potential, it has its own limitations. Diverse sensitivity and specificity of ctDNA are observed in most trials due to different techniques and platforms (139). In conclusion, how to standardize the detection procedures of existing biomarkers (such as PD-L1 and TMB) and how to search the new biomarkers to join the prediction team is a critical part. And the combination of multiple biomarkers, rather than a single molecule, will provide stronger value for immunotherapy-based precision treatment. In detail, the results from the CheckMate 026 trial has indicated that the OSR of those with TMB-high and PD-L1 ≥ 50% was higher than that of those with PD-L1 ≥ 50% alone (75 vs. 45.6%) (140). Similar studies focusing on digestive system cancers should be conducted in the future.

## Author Contributions

ZL contributed to the conception and idea of this article. ZZ and BY contributed to the literature search. ZZ wrote the manuscript. All authors have read and approved the final manuscript.

## Conflict of Interest

The authors declare that the research was conducted in the absence of any commercial or financial relationships that could be construed as a potential conflict of interest.
